# Simultaneous Rupture of an Aortomitral Intervalvular Fibrosa Pseudoaneurysm to the Aorta and the Left Atrium

**Published:** 2019-10

**Authors:** Reza Mohseni-Badalabadi, Ali Hosseinsabet, Mehdi Mohseni-Badalabadi, Khalil Forozannia

**Affiliations:** *Tehran Heart Center, Tehran University of Medical Sciences, Tehran, Iran.*

**Keywords:** *Heart atria*, *Aorta*, *False aneurysm*, *Echocardiography*

A 55-year-old man was admitted to our surgical ward with a diagnosis of an aorto-left atrial fistula. The diagnosis of infectious endocarditis had been ruled out in another hospital before the patient was referred to our hospital through multiple blood cultures, examinations of inflammatory markers, and the absence of signs and symptoms of infection. He had a history of aortic and mitral valve replacement with mechanical bileaflet valves 3 years earlier and had been on hemodialysis for several years. 

Physical examinations revealed a continuous murmur at the left parasternal border. Transthoracic and transesophageal echocardiographic examinations demonstrated mild left ventricular enlargement with a normal systolic function, a normal right ventricular size with systolic dysfunction, and normally functioning aortic and mitral prosthetic valves without any leakage or thrombosis. There was an echo-free space between the aortic and mitral valves that expanded in systole and decompressed in diastole with a connection to the left ventricular outflow tract, suggestive of the pseudoaneurysm of the aortomitral intervalvular fibrosa (AMIVF). This pseudoaneurysm was connected to the aorta on one side and to the left atrium on the other side, constituting a cavity between the ascending aorta and the left atrium that conducted a continuous flow from the aorta to the left atrium (Figure 1A and B; Video 1 and Video 2). Computed tomography angiography documented this space and its connection to the ascending aorta and the left atrium (Figure 2A, B, and C). It appears that surgical trauma was the most probable etiology of the pseudoaneurysm of the AMIVF in our patient. The patient refused surgical repair of this pseudoaneurysm. 

The AMIVF is a zone between the anterior mitral leaflet and the non-coronary and left coronary aortic cusp that is fibrotic and vascular. These properties predispose it to injury due to infection and surgical trauma as the most common etiologies for the pseudoaneurysm of the AMIVF. The pseudoaneurysm of the AMIVF can be complicated by fistulation to the adjacent structures such as the left atrium the and aorta, which has been reported in about 20% of patients with the pseudoaneurysm of the AMIVF.^1^

On the follow-up of patients with aortic or mitral valve replacement or both, the presence of a pseudoaneurysm in the AMIVF as an uncommon complication of such valve replacements should be considered at the time of physical examinations and echocardiography.

**Figure 1:**
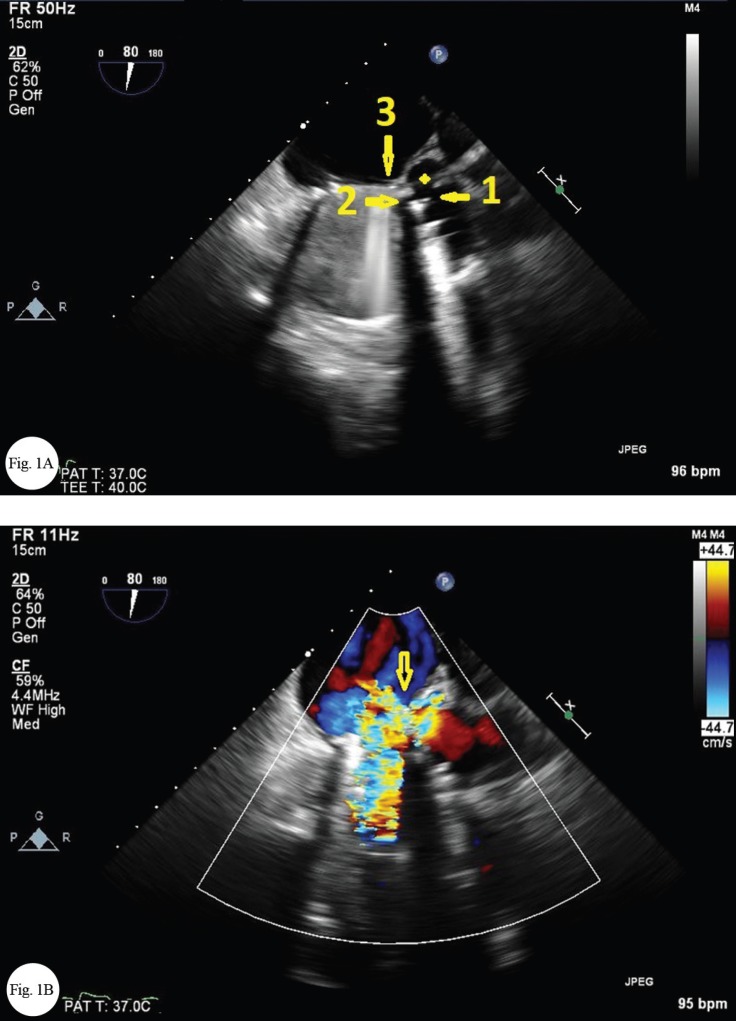
(A) Echo-free space between the mechanical mitral and aortic valves that expanded in systole and decompressed in diastole (4-point star), suggestive of a pseudoaneurysm in the aortomitral intervalvular fibrosa with a connection to the ascending aorta (arrow 1), the left ventricular outflow tract (arrow 2), and the left atrium (arrow 3) on transesophageal echocardiography. (B) Connection of the pseudoaneurysm of the aortomitral intervalvular fibrosa to the ascending aorta and the left atrium (arrow), resulting in a continuous flow through this pseudoaneurysm from the ascending aorta to the left atrium on transesophageal echocardiography

**Figure 2:**
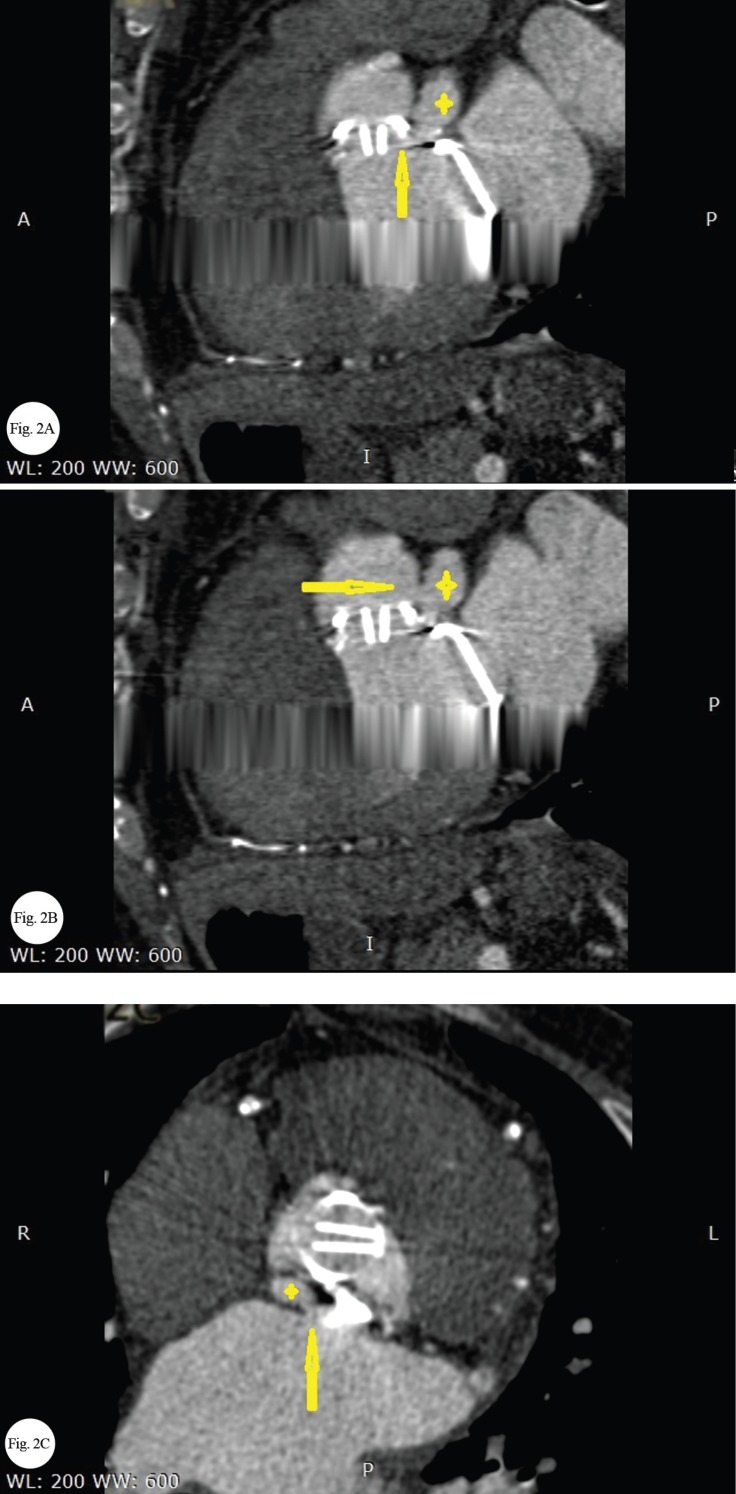
Cardiac computed tomography angiography, showing the pseudoaneurysm (4-point star) with (A) a connection to the left ventricular outflow tract (arrow), (B) the ascending aorta (arrow), and (C) the left atrium (arrow)

## Notes:

***This paper should be cited as: ***Mohseni-Badalabadi R, Hosseinsabet A, Mohseni-Badalabadi M, Forozannia K. Simultaneous Rupture of an Aortomitral Intervalvular Fibrosa Pseudoaneurysm to the Aorta and the Left Atrium. J Teh Univ Heart Ctr 2019;14(4):195-197.

## Notes:

***To watch the following videos, please refer to the relevant URLs:***

http://jthc.tums.ac.ir/index.php/jthc/article/view/1086/876

Video 1. Echo-free space between the mechanical mitral and aortic valves that expanded in systole and decompressed in diastole with a connection to the left ventricular outflow tract, suggestive of a pseudoaneurysm in the aortomitral intervalvular fibrosa on transesophageal echocardiography

http://jthc.tums.ac.ir/index.php/jthc/article/view/1086/877

Video 2. Connection of this pseudoaneurysm of the aortomitral intervalvular fibrosa to the ascending aorta and the left atrium, resulting in a continuous flow through this pseudoaneurysm from the ascending aorta to the left atrium on transesophageal echocardiography

## References

[1] Sudhakar S, Sewani A, Agrawal M, Uretsky BF (2010). Pseudoaneurysm of the mitral-aortic intervalvular fibrosa (MAIVF): A comprehensive review. J Am Soc Echocardiogr.

